# Burden and distribution of serologically detected HIV and syphilis infections among pregnant women attending antenatal care in peri-urban Blantyre, Malawi

**DOI:** 10.1371/journal.pgph.0006695

**Published:** 2026-06-22

**Authors:** Nathan Kawonga, Glory Kaunda, Innocent Machillika, Roselyn Chipojola, Flywell Kawonga

**Affiliations:** 1 School of Global and Public Health, Kamuzu University of Health Sciences, Blantyre, Malawi; 2 Malawi-Liverpool-Wellcome Programme, Queen Elizabeth Central Hospital, Blantyre, Malawi; 3 Institute of Infection, Veterinary & Ecological Sciences, Faculty of Health and Life Sciences, University of Liverpool, United Kingdom; 4 Department of Medical Laboratory Sciences, School of Life Sciences and Allied Health, Professions, Kamuzu University of Health Sciences, Blantyre, Malawi; PLOS: Public Library of Science, UNITED STATES OF AMERICA

## Abstract

Pregnant women are vulnerable to sexually transmitted infections (STIs), which can lead to adverse pregnancy outcomes. Evidence on STI prevalence and determinants among pregnant women in peri-urban Malawi remains limited. This study aimed to determine human immunodeficiency virus (HIV) and syphilis prevalence, identify associated factors and assess antenatal care (ANC) service quality among pregnant women in Blantyre peri-urban health facilities. A cross-sectional mixed-methods study was conducted at three peri-urban health centers (Zingwangwa, Ndirande, and Limbe). Quantitative data were retrospectively collected from facility records between January and June 2022 to determine STI diagnoses. Structured questionnaires assessed contributing factors and ANC service quality. Among 5,634 pregnant women tested for HIV, the prevalence of serologically detected HIV was 10.4% (95% confidence interval (CI): 9.7-11.3). Among 2,983 women tested for syphilis, the prevalence was 11.1% (95% CI: 10.0-12.3). The prevalence varied significantly by facility (p < 0.001), with the highest burden recorded at Limbe (HIV: 12.5%, 95% CI: 11.1-13.9; syphilis: 14.8%, 95% CI: 12.8-16.9), versus Ndirande (HIV: 10.5%, 95% CI: 9.1-12.1; syphilis: 8.4%, 95% CI: 6.7-10.3) and Zingwangwa (HIV: 7.5%, 95% CI: 6.2-8.9; syphilis: 8.9%, 95% CI: 7.0-11.0). Among women tested for both infections (n = 2,917), co-infection prevalence was 2.0% (95% CI: 1.6-2.6). Among 91 interviewed women (aged 14–43 years), unemployment (odds ratio (OR) 2.74, 95% CI: 1.07–7.03; p = 0.033) and lack of partner treatment (OR 2.83, 95% CI: 1.12–7.19; p = 0.025) were significantly associated with STI positivity. Although most participants were satisfied with ANC services, 64% reported that their partners were not treated, and no formal follow-up system existed, revealing gaps in partner management. The high HIV and syphilis prevalence highlights a pressing public health concern. Strengthening antenatal screening, improving partner management and implementing targeted interventions are essential to reduce HIV and syphilis burden among pregnant women in peri-urban Blantyre.

## Introduction

Sexually transmitted infections (STIs) are predominantly spread through unprotected sexual contact and are caused by bacteria, viruses, or parasites [1]. STIs remain a major public health concern, particularly in low- and middle-income countries, affecting individuals of all sexes and age groups, with the highest burden among people of reproductive age [2]. STIs can lead to substantial maternal and neonatal morbidity [3,4]. While over thirty infections can be sexually transmitted, the four most common curable non-viral infections include chlamydia, gonorrhea, trichomoniasis and syphilis caused by *Chlamydia trachomatis, Neisseria gonorrhoeae, Trichomonas vaginalis, and Treponema pallidum* respectively [5]. STIs increase the risk of HIV acquisition and transmission between two to eight fold [6,7]. In addition, the inflammation caused by viral and non-viral STIs can enhance viral shedding of HIV-1 in the genital tract which also increases the risk of HIV-1 transmission to the sex partners [7,8].

STIs are the second most common cause of unhealthy life among women aged 15–44 years in Africa [2]. Globally, approximately half a billion new cases occur each year and more than one million STIs are acquired per day [7]. Sub-Saharan Africa has an STI incidence of 240 per 1000 persons which is the highest in the world [8]. However, their prevalence varies widely across countries and among different subpopulations within the same country [3,9]. For instance, studies among pregnant women have reported overall STI prevalence of 53.6% in The Gambia [2], 17.5%, in Nepal [10], and 49.4% in Ilorin, Nigeria [11].

Symptoms of STIs include ano-genital ulcers, vaginal or urethral discharge, or lower abdominal pain, although the majority of infections remain asymptomatic [12,13]. In resource-limited settings, etiologic laboratory testing for curable STIs such as chlamydia and gonorrhea is often unavailable; therefore, the World Health Organization (WHO) recommends a syndromic approach for their management [14]. Under this approach, women presenting with vaginal discharge syndrome, lower abdominal pain syndrome or genital ulcer disease are treated with a combination of broad-spectrum antibiotics without waiting for laboratory confirmation [14]. In contrast, both WHO and Malawi national guidelines recommend universal testing for HIV and syphilis for all pregnant women at the first antenatal care visit as a standard component of prevention of mother-to-child transmission program [15].

Specific infections are associated with distinct adverse outcomes. Syphilis is a leading cause of stillbirth and can lead to severe congenital infection [9,16]. Bacterial STIs such as chlamydia and gonorrhea have been linked to preterm birth, low birth weight, and postpartum endometritis [9]. Furthermore, STIs collectively increase the risk of infertility, ectopic pregnancy, pelvic inflammatory disease, and puerperal sepsis [17]. HIV, in particular, has been strongly implicated in poor pregnancy outcomes in resource-limited settings due to its high prevalence in these settings [18,19].

Multiple studies have shown that risk of STIs during pregnancy is influenced by socio-demographic, behavioral patterns and characteristics of partnership [20,21]. In Papua New Guinea, higher prevalence was linked to having a partner perceived to be at risk, maternal extramarital intercourse, early sexual debut, lack of formal education, urban residence and smoking [22]. In Southern Mozambique, divorce or widowhood, multiple sexual partners and having a partner living far away were associated with higher risk of STIs [23]. Similarly, a study from Southern Ethiopia found that being unmarried status, age < 25 years, lower educational attainment and multiple sexual partners were linked to increased STI prevalence [20]. These studies demonstrate how personal behaviors, partnership patterns and broader social determinants may influence the risk of STIs during pregnancy.

Ensuring access to high-quality antenatal care remains vital in order to protect both maternal and fetal health [24]. A study conducted in fourteen rural health units in Tanta city in Egypt found that 94.3% of pregnant women were satisfied with the antenatal care received [25]. In The Gambia, satisfaction was 79.9% in public facilities and 97.9% in private facilities [26]. Negative perceptions of public facilities were linked to limited space, poor cleanliness, long waiting times and ineffective communication with healthcare workers [26].

Although STIs in pregnancy have been described in other sub-Saharan African settings, the prevalence and associated determinants among pregnant women in peri-urban Malawi are poorly characterised. Understanding local epidemiological patterns and etiological agents is essential for service planning, strengthening infection-control, improving partner management, ensuring timely diagnosis, and treatment. Therefore, this study was carried out to determine the serologically detected HIV and syphilis prevalence among pregnant women attending antenatal clinics in peri-urban health centers in Blantyre, identify factors associated with the STIs and evaluate the quality of antenatal care services.

## Methodology

### Study design and setting

We conducted a cross-sectional mixed-methods study with quantitative and qualitative components. The study was carried out at three peri-urban health centers in Blantyre, namely, Limbe, Zingwangwa, and Ndirande. The three health centers were purposively selected from the list of all 23 public health centers in the district as they are high-use peri-urban ANC facilities in Blantyre that provide integrated HIV and syphilis screening, and together they represent different peri-urban catchment populations, allowing comparison of the burden and distribution of infections across facilities.

### Study population

The study population comprised all pregnant women attending antenatal care (ANC) at the selected facilities between January and June 2022 who underwent serological testing for sexually transmitted infections, specifically HIV and syphilis. The six-month period was chosen to capture women across all trimesters of pregnancy [27]. The age of participants in the interviewed sample ranged from 14 to 43 years. Pregnant women who were unwilling to participate were excluded. In addition, participants with missing or undocumented HIV and/or syphilis results were excluded from analyses of serologically detected STIs.

### Laboratory procedures

HIV testing followed the Malawi national serial rapid testing algorithm. A non-reactive first test was recorded as HIV-negative. A reactive first test was followed by a second test using a new finger-prick. If the second test was reactive, a third test using a new finger-prick was performed, and a reactive third test was recorded as HIV-positive. Discordant results were managed according to the national algorithm, and the final result was recorded in the ANC register [15].

Syphilis testing was based on a reactive Venereal Disease Research Laboratory (VDRL) non-treponemal test recorded in the ANC register. Confirmatory treponemal testing was not available at the study facilities; therefore, a positive VDRL result alone represents a presumptive diagnosis of syphilis, in accordance with WHO guidelines (2017) [28].

### Sampling technique, sample size and data collection

Data were collected from two distinct sources. Secondary quantitative data were manually extracted by the study investigators during the month of September 2022 from handwritten ANC register records for all attendees during the study period. The extracted data covered variables including serologically detected STI diagnoses, specifically a reactive Venereal Disease Research Laboratory (VDRL) test result for syphilis and a positive rapid antibody HIV test, alongside syndromic STI diagnoses, and the trimester of diagnosis. To ensure accuracy, the entered data were cross-checked against the original registers by a second investigator, and any discrepancies were resolved through consensus. For the qualitative component, primary data were collected via structured questionnaires administered to a convenience sample of pregnant women who were approached regardless of whether they were attending their first ANC booking visit or follow-up appointment. This data collection exercise was carried out over a one-week study period in the same month of September, 2022. The use of a convenience sampling approach for participant enrollment was adopted from a related methodological design used in a Tanzanian study [29]. The target sample size for this component was calculated using the single proportion formula, assuming a 50% prevalence, a 95% confidence level (Z = 1.96) and a 5% margin of error. The use of 50% prevalence and marginal error of 5% was adopted from a similar study which was done in Anyigba, Nigeria [30]. This yielded a minimum sample of 90.25, which was rounded up to 91 participants. The structured questionnaire captured socioeconomic factors (occupation), health-related variables (partner treatment status), and participants’ satisfaction with ANC services.

### Data management and analysis

Data were analyzed using Microsoft Excel 2013 and R version 4.3.2 (R Core Team, Vienna, Austria). Descriptive statistics, including frequencies and percentages, were used to summarize categorical variables. A positive STI was defined as serologically detected HIV and/or reactive VDRL result. Women without recorded HIV or syphilis results were excluded from prevalence analyses. Due to the modest sample size from the questionnaire component (n = 91), only bivariable analyses were performed to identify factors associated with STI positivity.

### Ethical considerations

The ethical approval for the study was obtained from College of Medicine Research Ethics Committee (COMREC), Malawi (U.11/21/3495). The study was conducted in line with the principles of the Declaration of Helsinki. Written informed consent was obtained from all participants before any study procedures. For retrospective data extracted from ANC registers, all personal identifiers were excluded during data entry to create a de-identified dataset for analysis. Participant autonomy, privacy, and confidentiality were considered by using coded identifiers instead of names. All participants were informed of their right to withdraw at any time without facing any consequences.

### Reporting guidelines

This study was reported in accordance with the Strengthening the Reporting of Observational Studies in Epidemiology (STROBE) guidelines for cross-sectional studies.

## Results

### Participants and case distribution across sites

Between January and June 2022, a total of 5,700 pregnant women attended ANC at the three selected peri-urban health centers in Blantyre during the study period. The distribution across sites was: Limbe (n = 2,396; 42.0%), Ndirande (n = 1,681; 29.5%), and Zingwangwa (n = 1,623; 28.5%).

### HIV prevalence and testing coverage

HIV testing coverage was nearly universal across all sites, with 5,634 women tested, representing an overall coverage of 98.8%. Coverage was consistently high across sites: 97.6% at Limbe, 99.9% at Ndirande, and 99.6% at Zingwangwa. Among those tested, 588 women were HIV-positive, yielding an overall HIV prevalence of 10.44% (95% CI: 9.65–11.27). HIV prevalence varied significantly by health center (p < 0.001), with the highest prevalence observed in Limbe (12.45%; 95% CI: 11.12–13.88), followed by Ndirande (10.48%; 95% CI: 9.05–12.07) and Zingwangwa (7.49%; 95% CI: 6.23–8.92)

### Syphilis prevalence and testing coverage

Syphilis testing coverage was substantially lower than HIV testing coverage. Only 2,983 women (52.3%) were tested for syphilis, with site-specific coverage of 49.8% at Limbe, 57.6% at Ndirande, and 50.6% at Zingwangwa. Among those tested, 330 were VDRL-reactive, resulting in an overall syphilis prevalence of 11.06% (95% CI: 9.95–12.25). Syphilis prevalence also differed significantly across sites (p < 0.001), with the highest burden in Limbe (14.75%; 95% CI: 12.78–16.89), compared to Ndirande (8.37%; 95% CI: 6.70–10.28) and Zingwangwa (8.88%; 95% CI: 7.02–11.04). The difference in test coverage between HIV (98.8%) and syphilis (52.3%) was statistically significant (p < 0.001).

### Dual testing and HIV-syphilis co-infection

Dual testing for both HIV and syphilis was completed for 2,917 women, representing 51.2% of all ANC attendees. Among those tested for both infections, 59 women were identified as co-infected, yielding an overall co-infection prevalence of 2.02% (95% CI: 1.55–2.60). Co-infection prevalence varied significantly across sites (p < 0.001), with the highest prevalence in Limbe (3.00%; 95% CI: 2.08–4.17), followed by Ndirande (1.97%; 95% CI: 1.18–3.06) and Zingwangwa (0.74%; 95% CI: 0.27–1.59).

Reasons for missing HIV or syphilis test results were not systematically documented in the ANC registers; however, facility staff reported frequent VDRL test kit stock-outs during the study period, although these could not be quantified.

Overall and site-specific HIV and syphilis testing coverage and prevalence, including dual testing and co-infection, are summarized in Table 1.

**Table 1:** Summary of site-specific and total HIV and syphilis testing coverage and prevalence among ANC attendees in peri-urban Blantyre; P values are from chi-square tests, and co-infection analyses were restricted to women with both HIV and syphilis results available (n = 2,917).

Indicator	Limbe	Ndirande	Zingwangwa	Total	P value (across sites)
Total ANC attendees, N	2,396	1,681	1,623	5,700	–
HIV testing					
Tested for HIV, n	2,338	1,680	1,616	5,634	–
HIV test coverage, %	97.6	99.9	99.6	98.8	–
HIV-positive, n	291	176	121	588	–
HIV prevalence, % (95% CI)	12.45 (11.12-13.88)	10.48 (9.05-12.07)	7.49 (6.23-8.92)	10.44 (9.65-11.27)	<0.001
Syphilis testing					
Tested for syphilis, n	1,193	968	822	2,983	–
Syphilis test coverage, %	49.8	57.6	50.6	52.3	–
Syphilis-positive, n	176	81	73	330	–
Syphilis prevalence, % (95% CI)	14.75 (12.78-16.89)	8.37 (6.70-10.28)	8.88 (7.02-11.04)	11.06 (9.95-12.25)	<0.001
Dual testing					
Tested for both HIV and syphilis, n	1,135	967	815	2,917	–
Dual test coverage, %	47.4	57.5	50.2	51.2	–
HIV-syphilis co-infection, n	34	19	6	59	–
Co-infection prevalence, % (95% CI)	3.00 (2.08-4.17)	1.97 (1.18-3.06)	0.74 (0.27-1.59)	2.02 (1.55-2.60)	<0.001

### Distribution by trimester of serologically detected HIV and syphilis

**Fig 1:**
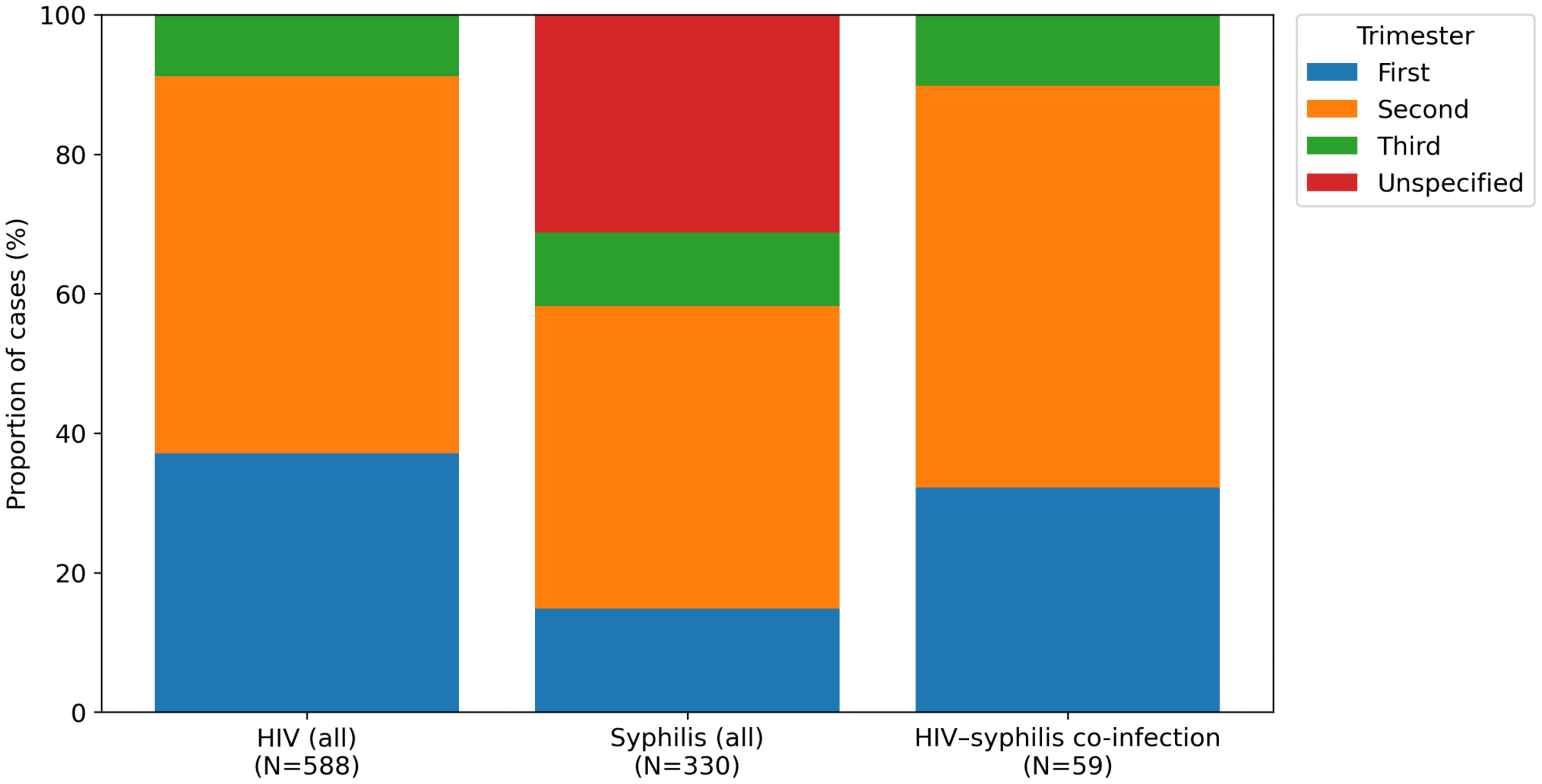
Distribution of serologically detected STI diagnoses by trimester. Percentages represent the proportion of diagnoses within each infection category occurring in each trimester. Distributions differed significantly by trimester for all infection categories (p < 0.001). Trimester-specific prevalence (i.e., the proportion infected among women tested in each trimester) could not be estimated because trimester-specific testing denominators were not available in the facility clinical records. These distributions therefore reflect the timing of diagnosis rather than true trimester-specific burden, as women may have been diagnosed at either their first or follow-up ANC visits.

The timing of serologically detected STI diagnoses differed significantly by trimester (p < 0.001), with most diagnoses recorded in the second trimester across all infection categories (Fig 1). Of the 588 women diagnosed with HIV, 218 (37.0%; 95% CI: 33.2–41.1) were identified in the first trimester, 318 (54.1%; 95% CI: 50.0–58.1) in the second trimester, and 52 (8.8%; 95% CI: 6.7–11.4) in the third trimester (p < 0.001). Among the 330 women diagnosed with syphilis, 49 (14.8%; 95% CI: 11.3–19.2) were identified in the first trimester, 143 (43.3%; 95% CI: 38.0–48.8) in the second trimester, and 35 (10.6%; 95% CI: 7.6–14.5) in the third trimester; timing was unspecified for 103 (31.3%; 95% CI: 26.3–36.5) in the clinical registers (p < 0.001). Among the 59 women with HIV–syphilis co-infection, 19 (32.2%; 95% CI: 21.0–45.6) were diagnosed in the first trimester, 34 (57.6%; 95% CI: 44.1–70.4) in the second trimester, and 6 (10.2%; 95% CI: 3.8–20.8) in the third trimester (p < 0.001).

### Distribution of syndromic diagnoses of STIs

**Fig 2:**
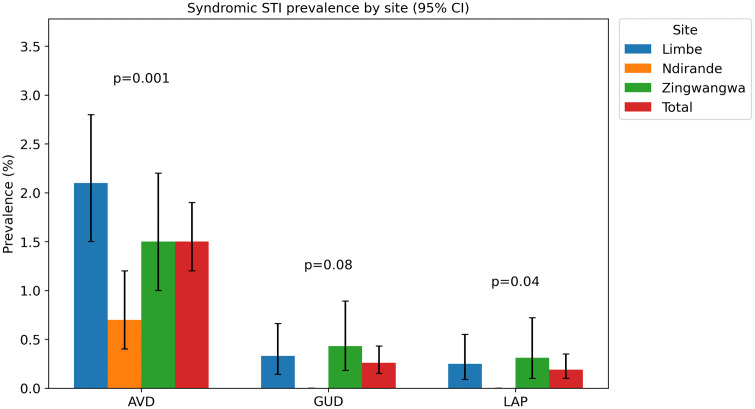
Proportion of pregnant women with syndromic STI diagnoses by facility.

Syndromic STI prevalence was calculated using all 5,700 ANC attendees as the denominator, since clinical diagnoses were made independently of laboratory testing availability. Abnormal vaginal discharge (AVD) was the most frequently recorded syndrome, diagnosed in 1.5% of women (95% CI: 1.2–1.9), followed by genital ulcer disease (GUD) in 0.26% (95% CI: 0.15–0.43) and lower abdominal pain (LAP) in 0.19% (95% CI: 0.10–0.35). Syndromic diagnoses varied significantly by facility. AVD prevalence was highest at Limbe (2.1%; 95% CI: 1.5–2.8) compared with Ndirande (0.7%; 95% CI: 0.4–1.2) and Zingwangwa (1.5%; 95% CI: 1.0–2.2) (p = 0.001). GUD was reported only at Limbe (0.33%) and Zingwangwa (0.43%), with no cases recorded at Ndirande (p = 0.08). LAP also varied by facility (p = 0.04). Site-specific syndromic prevalence is shown in Fig 2.

### Factors associated with STI prevalence

Of the 91 interviewed women, 27 (29.7%) were STI-positive. In bivariable analysis, unemployment and inadequate partner treatment were significantly associated with STI positivity. Compared with women who were employed or in business, unemployed women had 2.74-fold higher odds of STI positivity (18/45 vs 9/46; OR 2.74, 95% CI: 1.07–7.03; p = 0.033). Similarly, women whose partners were not treated also had higher odds of being positive (17/41 vs 10/50; OR 2.83, 95% CI: 1.12–7.19; p = 0.025). Factors associated with STI positivity are presented in Table 2.

**Table 2:** Bivariable logistic regression analysis of factors associated with STI positivity among interviewed pregnant women attending ANC (n = 91).

Characteristic	Category	STI + n/N (%)	OR (95% CI)	p-value
Occupation	Employed/Business	9/46 (19.6)	Ref	
	Unemployed	18/45 (40.0)	2.74 (1.07–7.03)	0.033
Partner treatment	Treated	10/50 (20.0)	Ref	
	Not treated	17/41 (41.5)	2.83 (1.12–7.19)	0.025

Abbreviations: STI, sexually transmitted infection; n, number of STI-positive participants; N, total number of participants in each category; OR, odds ratio; CI, confidence interval

### Patient satisfaction and health service quality

When asked about their experience with ANC services, 55 women (60.4%) reported being very satisfied (95% CI: 49.9–70.0), 9 (9.9%) were satisfied (95% CI: 5.2–17.9) and nearly a third (29.7%) expressed dissatisfaction (95% CI: 21.3–39.8). Although the majority expressed high satisfaction, the substantial minority reporting poor satisfaction indicates notable gaps in service delivery that warrant programmatic attention at ANC services. Pregnant women’s satisfaction with ANC services is shown in the Table 3.

**Table 3:** Satisfaction with ANC services among interviewed pregnant women (n = 91).

Satisfaction level	n (%) [95% CI]
**Very satisfied**	55 (60.4) [49.9–70.0]
**Satisfied**	9 (9.9) [5.2–17.9]
**Dissatisfied**	27 (29.7) [21.3–39.8]
**Total**	91 (100.0)

## Discussion

This multi-facility study in peri-urban Blantyre has shown a significant burden of STIs among pregnant women attending ANC and reveals considerable variation across the three sites. The observed HIV prevalence was 10.4% (95% CI: 9.7–11.3), while syphilis prevalence was 11.1% (95% CI: 10.0–12.3), with Limbe consistently recording the highest burden. Comparison with recent data from Queen Elizabeth Central Hospital (QECH), the main referral hospital in the same city, reveals important epidemiological heterogeneity [16]. Our finding of lower HIV prevalence (10.4% vs. 16.5% at QECH) may reflect the different patient populations, as the tertiary facility likely attracts more women with known HIV or complex pregnancies. Conversely, the higher syphilis burden in peri-urban clinics (11.1% vs. 8.1%), coupled with our finding of critically low screening coverage (52.3%), highlights a potential syndemic in these settings driven by health system gaps. This disparity underscores that STI burdens are not uniform across service levels and that strengthening screening and treatment in peri-urban primary care is essential for equitable prevention and treatment measures.

These prevalence figures for specific infections place peri-urban Blantyre within the wide spectrum reported across sub-Saharan Africa and South Asia. Our HIV prevalence (10.4%) is consistent with national estimates, yet lower than overall STI prevalence in high-burden West African settings like The Gambia (53.6%), Ilorin, Nigeria (49.4%), and parts of Kenya (20.8%) [2,11,31]. The strikingly similar syphilis prevalence (11.1%) alongside HIV indicates a comparable epidemic pattern in this peri-urban context. This reflects the well-documented evidence that spatial clustering of STIs is influenced by population risk, sexual networks, and service organization [32,33].

The timing of STI detection is programmatically significant. Positive cases clustered in the second trimester for serologically detected STIs (HIV and syphilis) and syndromic presentations, with comparatively fewer diagnoses in the third-trimester. In Malawi, early ANC attendance remains suboptimal, with the majority of women initiating ANC services in fourth or fifth month of pregnancy, and re-testing later in pregnancy is often inconsistent [34]. Delayed or missed screening is a significant concern, given the established evidence that curable STIs such as Chlamydia, gonorrhea, trichomoniasis and syphilis are associated with adverse outcomes such as miscarriage, stillbirth, prematurity, low birth weight and postpartum infection [9].

The pathogen profile and testing practices help explain the pattern of prevalence we observed in our study. HIV testing was done on nearly all clients, whereas syphilis screening was inconsistent due to frequent stock-outs of VDRL test kits, leaving nearly half of pregnant women untested. This suggests an underestimation of true syphilis prevalence and complicates comparisons across facilities. A recent tertiary-hospital report from Blantyre, where etiologic testing was available, found lower gonorrhea (3.1%) and chlamydia (2.7%) prevalence, but similar HIV and syphilis burdens [16], illustrating how diagnostic approach and service level shape the measured prevalence.

Co-infection with HIV and syphilis was identified in about 2% of tested women, representing a smaller yet important high-risk subgroup. Biological and epidemiologic evidence indicate that ulcerative and inflammatory STIs, including syphilis raise HIV acquisition and transmission two- to eight-fold and increase genital HIV-1 shedding, increasing maternal–fetal and sexual transmission risks [6,8]. In pregnancy, HIV and syphilis are each independently associated with stillbirth, prematurity, low birth weight, and postpartum infection. When co-occurring, they further increase the risk of adverse birth outcomes and congenital syphilis [9]. Regional data show wide variation of co-infection, with markedly higher prevalence in Botswana (37.7% of women with syphilis were HIV-positive, with excess adverse outcomes) and lower levels in Luanda, Angola (5% of HIV-positive women co-infected with syphilis) [35,36]. Our lower prevalence compared to other countries may represent an underestimation due to frequent stock-outs of VDRL test kits and incomplete syphilis screening, which would bias co-infection downward. Programmatically, even at low levels, these findings support the need for integrated antenatal screening, universal HIV and syphilis testing at first ANC with re-testing later in pregnancy, reliable dual-rapid/Treponemal test supply and immediate treatment together with partner notification/treatment to break reinfection cycles [37,38]. Effective counselling on congenital syphilis prevention and linkage to follow-up care remain paramount to alleviate the risks of dual infection.

Syndromic data offered useful clinical insight but also underlined the limitations of syndromic management in pregnancy. For instance, AVD, the most common syndromic diagnosis varied significantly by site; serologically detected infections were more frequently identified in the second trimester whereas genital ulcer disease and lower abdominal pain were less frequent but showed similar pattern. These findings are consistent with studies from other low- and middle-income (LMICs) settings [39,40]. As many infections in pregnancy are asymptomatic and syndromic algorithms have low sensitivity and specificity, reliance on syndromic care risks both missed infections and unnecessary treatment which may lead to antimicrobial resistance as well [9,41–43]. Where feasible, etiologic testing should complement syndromic approach to improve diagnostic accuracy, enhance appropriate treatment and support partner management.

The two factors associated with STI positivity in the bivariable logistic regression were actionable. For instance, unemployment was associated with nearly three-fold higher odds of STI positivity compared with employment or business (OR 2.74, 95% CI: 1.07–7.03). This aligns with evidence that socioeconomic disadvantage heightens the risk of STI acquisition by constraining personal agency, increasing exposure to transactional sex and limiting access to prevention and care [20,21,44,45]. Inadequate partner treatment was also associated with higher odds of STI positivity (OR 2.83, 95% CI: 1.12–7.19), highlighting regional evidence that partnership dynamics, including partner mobility, concurrency and non-treatment, put women at risk [46].

Women’s experience of ANC services was mixed, with 60.4% reported being very satisfied and a further 9.9% were satisfied, and comparable to reports from Ethiopia and some Nigerian tertiary settings and slightly below private-sector estimates from The Gambia [26,47,48]. Nearly one-third were not satisfied. Feedback from the interviews pointed to limited STI screening and inadequate partner treatment, reflecting broader evidence that perceived client satisfaction can exist even when service-delivery falls short in areas like space, cleanliness, waiting times and effective communication [26,49]. These findings emphasize the need for patient-centered ANC that integrates dependable diagnostics, proper partner management and clear communication about testing and follow-up.

Some notable limitations are acknowledged in this study. Some participants lacked prior health passports, constraining historical clinical data. Except for HIV and syphilis, diagnoses relied on syndromic assessment and trimester-specific denominators were often missing, precluding true trimester-specific prevalence estimates. Some women did not have recorded serologically detected HIV and/or syphilis results and were therefore not included in the prevalence analysis; together with intermittent stock-outs of test kits, this may limit the generalizability of our STI prevalence estimates. This also raises the possibility of selection bias if clinicians preferentially tested symptomatic women at the facilities. Furthermore, our prevalence estimates and statistical tests did not account for clustering of participants within health facilities, which may have led to a slight underestimation of variance. The primary data were collected from a convenience sample of women attending ANC during a one-week period, which included both first and follow-up visits. This may affect the generalizability of qualitative findings regarding partner treatment status and satisfaction, as women at different stages of ANC may have different experiences. Risk-factor analyses from the interviews were unadjusted and may be confounded by unmeasured variables.

Despite these limitations, the findings of the study provide clear programmatic guidance consistent with WHO’s vision for high-quality ANC. Ensuring HIV and syphilis testing at first ANC and re-testing in pregnancy, strengthening partner notification and treatment, including patient-delivered partner therapy [46], and reinforcing comprehensive risk-reduction counselling strategies, remain essential components of ANC-based STI control programs. Complementing syndromic management with etiologic testing where possible and improving data capture (test performed/not performed, trimester and partner-treatment status) will support accurate monitoring and quality improvement. Together, these actions have the potential to reduce preventable maternal and neonatal morbidity in peri-urban Malawi and to support progress toward national and global sexual and reproductive health goals.

In summary, this study underscores the high burden of STIs among pregnant women in peri-urban Blantyre and identifies programmatic gaps that should be addressed. Strengthening ANC services through universal screening and re-testing, ensuring a steady supply of test kits, implementing routine partner notification and treatment, improving clinical documentation and focusing targeted prevention strategies on socioeconomically vulnerable women are essential.

## Supporting information

S1 File STROBE checklist for cross-sectional studies. A completed checklist indicating where each item of the STROBE guidelines is addressed in the manuscript. The STROBE checklist is reproduced from the STROBE Statement and distributed under the CC BY 4.0 license. Source: https://www.strobe-statement.org/.
(DOCX)
