# Supplementary material for: Burden and distribution of serologically detected HIV and syphilis infections among pregnant women attending antenatal care in peri-urban Blantyre, Malawi
Source: PLOS Glob Public Health. 2026 Jun 22;6(6):e0006695. doi: 10.1371/journal.pgph.0006695 (PMC13286208; doi:10.1371/journal.pgph.0006695)
Supplement: S1 File — A completed checklist indicating where each item of the STROBE guidelines is addressed in the manuscript. The STROBE checklist is reproduced from the STROBE Statement and distributed under the CC BY 4.0 license. Source: https://www.strobe-statement.org/. (DOCX) [file pgph.0006695.s001.docx]

# S1 STROBE Checklist for Cross-Sectional Studies

**Manuscript:** Burden and distribution of serologically detected HIV and syphilis infections among pregnant women attending antenatal care in peri-urban Blantyre, Malawi.

The STROBE checklist is reproduced from the STROBE Statement and distributed under the CC BY 4.0 license. Source: <https://www.strobe-statement.org/>

| **Section** | **Item No.** | **STROBE Recommendation** | **Relevant text from revised manuscript** | **Manuscript Section** | **Page No(s).** |
| --- | --- | --- | --- | --- | --- |
| Title & Abstract | 1a | Indicate the study’s design with a commonly used term in the title or the abstract. | A cross-sectional mixed-methods study was conducted at three peri-urban health centers of Blantyre (Zingwangwa, Ndirande, and Limbe). | Abstract | p.1-2 |
| Title & Abstract | 1b | Provide in the abstract an informative and balanced summary of what was done and what was found. | Quantitative data were retrospectively collected from facility to determine STI diagnoses. Structured questionnaires assessed contributing factors and ANC service quality. Among 5,634 pregnant women tested for HIV, the prevalence of serologically detected HIV was 10.4% (95% CI: 9.7–11.3). Among 2,983 women tested for syphilis, the prevalence was 11.1% (95% CI: 10.0–12.3). | Abstract | p.2 |
| Introduction | 2 | Explain the scientific background and rationale for the investigation being reported. | Although STIs in pregnancy have been described in other sub-Saharan African settings, the prevalence and associated determinants among pregnant women in peri-urban Malawi are poorly characterised. Understanding local epidemiological patterns and etiological agents is essential for service planning, strengthening infection-control, improving partner management, ensuring timely diagnosis, and treatment. | Introduction | pp.5-6 |
| Introduction | 3 | State specific objectives, including any prespecified hypotheses. | Therefore, this study was carried out to determine the serologically detected HIV and syphilis prevalence among pregnant women attending antenatal clinics in peri-urban health centers in Blantyre, identify factors associated with the STIs and evaluate the quality of antenatal care services. | Introduction | p.5-6 |
| Methods | 4 | Present key elements of study design early in the paper. | We conducted a cross-sectional mixed-methods study with quantitative and qualitative components. | Methods | p.6 |
| Methods | 5 | Describe the setting, locations, and relevant dates, including periods of recruitment, exposure, data collection, and follow-up. | The study population comprised all pregnant women attending antenatal care (ANC) at the selected facilities between January and June 2022. | Methods | pp.6 |
| Methods | 6a | Give the eligibility criteria, and the sources and methods of selection of participants. | The study population comprised all pregnant women attending antenatal care (ANC) at the selected facilities between January and June 2022 who underwent laboratory-confirmed testing for sexually transmitted infections, specifically HIV and syphilis. | Methods | pp.6 |
| Methods | 6b | For matched studies, give matching criteria and number of exposed and unexposed. | Not applicable (no matching was performed). |  | N/A |
| Methods | 7 | Clearly define all outcomes, exposures, predictors, potential confounders, and effect modifiers. Give diagnostic criteria, if applicable. | A positive STI was defined as serologically detected rapid antibody HIV and/or reactive VDRL test. | Methods | pp.8 |
| Methods | 8 | For each variable of interest, give sources of data and details of methods of assessment (measurement). Describe comparability of assessment methods if there is more than one group. | Secondary quantitative data were extracted from antenatal care registers, while primary data were collected using structured questionnaires administered to pregnant women attending ANC. | Methods | p.7 |
| Methods | 9 | Describe any efforts to address potential sources of bias. | This also raises the possibility of selection bias if clinicians preferentially tested symptomatic women at the facilities. | Discussion | p.11 |
| Methods | 10 | Explain how the study size was arrived at. | The sample size was calculated using a single population proportion formula, assuming an estimated STI prevalence of 50%, a 95% confidence level, and a 5% margin of error. | Methods | pp.7 |
| Methods | 11 | Explain how quantitative variables were handled in the analyses. If applicable, describe which groupings were chosen and why. | Descriptive statistics, including frequencies and percentages, were used to summarize categorical variables | Methods | Pp.8 |
| Methods | 12a | Describe all statistical methods, including those used to control for confounding. | bivariable logistic regression analysis was conducted to assess factors associated with STI positivity. | Methods | pp.8 |
| Methods | 12b | Describe any methods used to examine subgroups and interactions. | Prevalence estimates were stratified by facility and by trimester of pregnancy. | Methods | p.6-7 |
| Methods | 12c | Explain how missing data were addressed. | Participants with missing or undocumented laboratory-confirmed HIV and/or syphilis results were excluded from prevalence analyses. | Methods | p.8 |
| Methods | 12d | If applicable, describe analytical methods taking account of sampling strategy. | Our prevalence estimates and statistical tests did not account for clustering of participants within health facilities, which may have led to a slight underestimation of variance. | Discussion | p.21 |
| Methods | 12e | Describe any sensitivity analyses. | Not applicable (no sensitivity analyses were conducted). |  | N/A |
| Results | 13a | Report numbers of individuals at each stage of study (e.g., numbers potentially eligible, examined for eligibility, confirmed eligible, included in the study, completing follow-up, and analysed). | Between January and June 2022, a total of 5,700 pregnant women attended ANC at the three selected peri-urban health centers in Blantyre during the study period. | Results | pp.9 |
| Results | 13b | Give reasons for non-participation at each stage. |  |  | N/A |
| Results | 13c | Consider use of a flow diagram. | Not applicable (a flow diagram was not used). |  | N/A |
| Results | 14a | Give characteristics of study participants (e.g., demographic, clinical, social) and information on exposures and potential confounders. | In bivariable analysis, unemployment and inadequate partner treatment were significantly associated with STI positivity | Results | pp.15 |
| Results | 14b | Indicate number of participants with missing data for each variable of interest. |  |  | N/A |
| Results | 15 | Report numbers of outcome events or summary measures. | Among 5,634 pregnant women tested for HIV, the prevalence of serologically detected HIV was 10.4% (95% CI: 9.7-11.3). Among 2,983 women tested for syphilis, the prevalence was 11.1% (95% CI: 10.0-12.3) | Results | pp.9–11 |
| Results | 16a | Give unadjusted estimates and, if applicable, confounder-adjusted estimates and their precision (e.g., 95% confidence interval). | Unemployed women had higher odds of STI positivity compared to employed women (OR 2.74, 95% CI: 1.07–7.03). | Results | pp.14-15 |
| Results | 16b | Report category boundaries when continuous variables were categorized. |  |  | N/A |
| Results | 16c | If relevant, consider translating estimates of relative risk into absolute risk for a meaningful time period. | Not applicable. |  | N/A |
| Results | 17 | Report other analyses done (e.g., analyses of subgroups and interactions, and sensitivity analyses). | Analyses were also conducted to describe the distribution of serologically detected HIV and syphilis by trimester and by health facility. | Results | pp.12–14 |
| Discussion | 18 | Summarise key results with reference to study objectives. | This multi-facility study in peri-urban Blantyre has shown a significant burden of STIs among pregnant women attending ANC and reveals considerable variation across the three sites | Discussion | p.16 |
| Discussion | 19 | Discuss limitations of the study, taking into account sources of potential bias or imprecision. Discuss both direction and magnitude of any potential bias. | Limitations include incomplete laboratory records, lack of adjustment for clustering by facility, and reliance on syndromic diagnosis for non-HIV, non-syphilis STIs. | Discussion | pp.20–21 |
| Discussion | 20 | Give a cautious overall interpretation of results considering objectives, limitations, multiplicity of analyses, results from similar studies, and other relevant evidence. | Except for HIV and syphilis, diagnoses relied on syndromic assessment and trimester-specific denominators were often missing, precluding true trimester-specific prevalence estimates. Some women did not have recorded serologically detected HIV and/or syphilis results and were therefore not included in the prevalence analysis; together with intermittent stock-outs of test kits, this may limit the generalizability of our STI prevalence estimates | Discussion | pp.20–21 |
| Discussion | 21 | Discuss the generalisability (external validity) of the study results. | [Interpretation contextualizes findings within other settings] | Discussion | p.16-22 |
| Other information | 22 | Give the source of funding and the role of the funders for the present study and, if applicable, for the original study on which the present article is based. | This study was funded by Kamuzu University of Health Sciences. The funders had no role in study design, data collection, analysis, interpretation, or manuscript preparation. | Funding |  |
